# A Performance Comparison between Different Industrial Real-Time Indoor Localization Systems for Mobile Platforms

**DOI:** 10.3390/s24072095

**Published:** 2024-03-25

**Authors:** Paulo M. Rebelo, José Lima, Salviano Pinto Soares, Paulo Moura Oliveira, Héber Sobreira, Pedro Costa

**Affiliations:** 1 Institute for Systems and Computer Engineering, Technology and Science (INESC TEC), 4200-465 Porto, Portugal; jllima@ipb.pt (J.L.); oliveira@utad.pt (P.M.O.); heber.m.sobreira@inesctec.pt (H.S.); pedrogc@fe.up.pt (P.C.); 2 School of Sciences and Technology-Engineering Department (UTAD), 5000-801 Vila Real, Portugal; salblues@utad.pt; 3 CeDRI, SusTEC, Instituto Politécnico de Bragança, Campus Sta Apolónia, 5300-253 Bragança, Portugal; 4 Institute of Electronics and Informatics Engineering of Aveiro (IEETA), University of Aveiro, 3810-193 Aveiro, Portugal; 5 Intelligent Systems Associate Laboratory (LASI), University of Minho, 4800-058 Guimarães, Portugal; 6 Faculty of Engineering, University of Porto (FEUP), 4200-465 Porto, Portugal

**Keywords:** indoor localization systems, localization technologies, sensors, autonomous mobile robots

## Abstract

The flexibility and versatility associated with autonomous mobile robots (AMR) have facilitated their integration into different types of industries and tasks. However, as the main objective of their implementation on the factory floor is to optimize processes and, consequently, the time associated with them, it is necessary to take into account the environment and congestion to which they are subjected. Localization, on the shop floor and in real time, is an important requirement to optimize the AMRs’ trajectory management, thus avoiding livelocks and deadlocks during their movements in partnership with manual forklift operators and logistic trains. Threeof the most commonly used localization techniques in indoor environments (time of flight, angle of arrival, and time difference of arrival), as well as two of the most commonly used indoor localization methods in the industry (ultra-wideband, and ultrasound), are presented and compared in this paper. Furthermore, it identifies and compares three industrial indoor localization solutions: Qorvo, Eliko Kio, and Marvelmind, implemented in an industrial mobile platform, which is the main contribution of this paper. These solutions can be applied to both AMRs and other mobile platforms, such as forklifts and logistic trains. In terms of results, the Marvelmind system, which uses an ultrasound method, was the best solution.

## 1. Introduction

To be more competitive, flexible, and productive, nowadays, all companies are modeling and investing in their factory floors. The industry is experiencing a new era of high technological development, so the integration of mobile platforms in industrial processes and tasks is increasingly common. Currently, all companies are focused on developing industrial systems that are fully automated and more flexible [1]. This flexibility makes them suitable to be used in different industrial stages or environments.

Autonomous guided vehicles (AGV) are normally mobile platforms to transport materials between workstations or warehouses, without guidelines like the magnetic lines, and their increased use in shop floors is related to their robustness and flexibility [2], contributing to the increase of efficiency and effectiveness of the production process.

In modern industries, AMR systems are a very attractive solution to increase the level of automation in factory logistics [3], so the use of them has become widespread in the last few decades.

The latest and most modern industries have integrated a hybrid system of mobile platform types on their factory floors. Manual platforms, such as logistic trains and forklift trucks, continue to perform their tasks, but with the help of autonomous mobile platforms, the well-known mobile robots. To have a good interaction between both, especially in path management, all these platforms must know their position and orientation so that the movement is safe and smooth [4], this is the main goal of the localization systems.

Indoor localization is a key technology for mobile platforms (MP) [5], such as AMR, as it enables the platform to determine its position and orientation within an indoor environment. There are several approaches to indoor localization, each with its own set of advantages and disadvantages.

As another important point, there are various industrial environments and their characteristics that must be taken into consideration to have an efficient localization robot system. Sometimes, a common solution is to use more than one of the technologies mentioned above and apply a sensor fusion algorithm.

The exchange between localization systems in real time, and consequently, the exchange of maps and trajectories, allows the robot to obtain greater stability in estimating its pose as well as in smoothing its movement on the factory floor. These data, of AMRs’ pose, are also very important for the robot fleet management algorithm.

This study is a module of a project aiming to integrate autonomous mobile robots (AMR) within logistical trains. To effectively plan the routes for the AMR, it is imperative to have accurate information about the positions of the logistic trains.

Considering the previous assumptions, this paper discusses the integration, in an industrial environment, and the comparison of different indoor localization systems for mobile platforms. The main goal is the selection of a real-time location system to be implemented in all mobile platforms of an industrial production line. The detection of the real location of each vehicle will allow the AMR fleet management software to plan more efficiently and accurately the AMRs paths, reducing conflicts between different mobile platforms on the shop floor (AMRs, forklifts, logistical trains, others).

In Section 2, the state-of-the-art indoor localization systems, technologies, and techniques are presented. Section 3 describes the most popular localization technologies and techniques usually implemented in indoor environments for locating objects and/or persons. Section 4 presents the systematic framework employed to address the research objectives. Section 5 presents the comparison results achieved with different indoor localization systems, in an industrial scenario and with an AMR. In this specific section, it is possible to compare and analyze the results obtained from the different industrial localization systems and their comparison with ground truth. Finally, some conclusions and the contribution of this research are presented in Section 6.

This paper stands out from the vast majority of papers in the literature on indoor localization, as it compares three industrial systems on the market in a quantitative way, with tests carried out in a real environment, making it easier to choose for future integration.

## 2. Related Work

Indoor localization refers to the process of determining the location of a device or a person inside a building or an enclosed space [6]. It is an important technology that has numerous applications in various industries and sectors, including retail, healthcare [7], transportation [8], industrial automation [9], public safety [10], and entertainment [11].

The importance of indoor localization lies in the fact that it enables businesses and organizations to better understand and optimize the movement and behavior of people and assets within their premises [12]. For example, indoor localization can help a retailer track customer movement and engagement in its store, a healthcare facility monitor the location and status of its medical equipment and staff, a transportation company optimize the delivery of packages and goods [13], and a factory automate [14] and monitor the production process [15].

There are different types of indoor localization systems, each using various technologies and methods to determine the location of a device or a person. Some common technologies used in indoor localization include radio frequency (RF) signals, wireless technologies (e.g., WiFi, Bluetooth), ultrasonic (US) signals, and infrared (IR) signals. Geometric methods [16] such as trilateration and triangulation are often used to calculate the position of a device based on the distance to multiple reference points [17]. Probabilistic methods such as Kalman filters and particle filters are also used to estimate the location based on statistical models and sensor data [14,18].

Regarding AMRs, nowadays, in industry, some technologies can be applied for autonomous mobile platforms localization like the indoor/outdoor Global Positioning System (GPS) [19,20], 2D/3D sensors, vision systems, and wireless technologies like radio frequency identification (RFID) tags [21,22] or barcodes [23,24,25]. They have different characteristics, namely the accuracy, and therefore, it is important to take into account the purpose of each robot.

In recent years, machine learning (ML) techniques [26,27,28,29] have also been applied to indoor localization to improve the accuracy and adaptability of the systems.

Concerning the transportation and logistics sectors, indoor localization systems can be used in warehouses, distribution centers, and other transportation and logistics environments to track the movement and status of packages [30], vehicles, and personnel. This can improve the efficiency and accuracy of package delivery [31] and inventory management [32], as well as reducing the risk of accidents and errors. For example, an indoor localization system can help a warehouse worker locate a specific package or pallet more quickly, or alert a driver when they are approaching a restricted area.

In industrial automation and smart factories, this type of localization system can be used in factories and other industrial environments to automate and monitor the production process. This can improve the efficiency, quality, and safety of manufacturing operations [33], as well as enable the use of advanced technologies such as robotics [34] and the Internet of Things (IoT) [35]. For example, an indoor localization system can be used to track the location and status of manufacturing equipment and materials, or to guide autonomous vehicles and robots through the factory [18].

One common approach is to use a fixed infrastructure, such as a network of stationary beacons or sensors [36,37], to determine the platform’s position. The platform can use these beacons or sensors to triangulate its position based on the strength of the signals it receives from each beacon [17]. This approach is relatively simple and accurate, but it requires the installation and maintenance of a fixed infrastructure, which may not be practical in all situations [36].

Another approach is to use computer vision techniques [38] to localize the robot or other platform. This can involve using visual features such as roofs [39], corners [40], edges, textures, or fiducial markers [41] in the environment to determine the robot’s position and orientation. This approach is generally more flexible and can work in a variety of environments, but it may be less accurate than other methods, particularly in cluttered or poorly lit environments.

Another option is to use Inertial Measurement Units (IMUs) to determine the platform’s position and orientation [42]. IMUs are sensors that measure acceleration and angular velocity and can be used in mobile robotics to track platform movement over time when integrated with other robot localization systems. This approach is relatively simple and can work in a variety of environments, but it may be prone to drift over time when used standalone, leading to errors in the platform’s position estimates [43].

Indoor Global Positioning System (GPS) systems are specialized versions of the GPS and are designed to work in indoor environments, where traditional GPS signals may be weak or unavailable [44]. These systems use a combination of technologies, such as Wi-Fi, Bluetooth, or ultra-wideband (UWB), to determine the location of a device or platform within an indoor space [45]. These systems are generally more accurate than a traditional GPS when used in indoor environments [46], but their accuracy can vary depending on the specific technologies and infrastructure used.

Finally, some indoor localization systems use a combination of these approaches, combining the strengths of different methods to achieve the best possible accuracy and flexibility [45]. For example, a robot may use a fusion algorithm to improve its accuracy and robustness in different environments [47]. However, all these types of localization systems, techniques, and methods are always subject to propagation problems and reflections of the signal itself, culminating in delays in its detection and subsequent errors in locating the object or person. In the chapter on data analysis, Section 5, it will be possible to validate these phenomena in the different indoor localization systems tested.

To the authors’ knowledge, so far, there is no article with the practical and implemented comparison of localization systems as highlighted in this article.

## 3. Localization Techniques and Methods

This section is divided into two subsections. The first discusses some of the most commonly used methodologies for obtaining the position of a given object or person, in different environments and in real time. The second subsection lists, describes, and characterizes some of the technologies that can be found in localization systems.

### 3.1. Localization Techniques

Several different methods can be used for indoor localization, each with its strengths and limitations. These methods can be broadly classified into three categories: Trilateration, Fingerprinting, and Dead Reckoning. This subsection will present three methods—time of flight (ToF), angle of arrival (AoA), and time difference of arrival (TDoA)—of the trilateration category.

#### 3.1.1. Time of Flight (ToF)

ToF, or time of arrival (ToA), is a method for measuring the distance between two radio transceivers [48]. It uses the signal propagation time ($\vec{\Delta }t$), between the transmitter (Tx) and the receiver (Rx), to determine the distance between them. The ToF value multiplied by the signal velocity (*v*) provides the physical distance ($D_{ij}$) between Tx and Rx (see Equation (1)).

(1) $$\begin{array}{cc} D_{ij} & =(t_{2}-t_{1})\times v\\ \; & =\vec{\Delta }t\times v \end{array}$$

where $t_{1}$ is the time when Tx, in pose *i*, sends a message to the Rx, in pose *j*. The last receives the signal at $t_{2}$, where $t_{2}$ = $t_{1}$ + $\vec{\Delta }t$ ($\vec{\Delta }t$ is the time taken, by the signal, between Tx and Rx). So, the distance between the *i* and *j*, $D_{ij}$, can be calculated by Equation (1), where *v* represents the speed of the signal.

The principal requirement of the ToF method is the synchronization between transmitters and receivers. The signal bandwidth and the sampling rate affect the system accuracy, where a low sampling rate (in time) reduces the ToF resolution. In industrial indoor environments, this type of method may have significant localization errors caused by the obstacles, that deflect the emitted signals from the transmitter to the receiver.

#### 3.1.2. Angle of Arrival (AoA)

**Figure 1:**
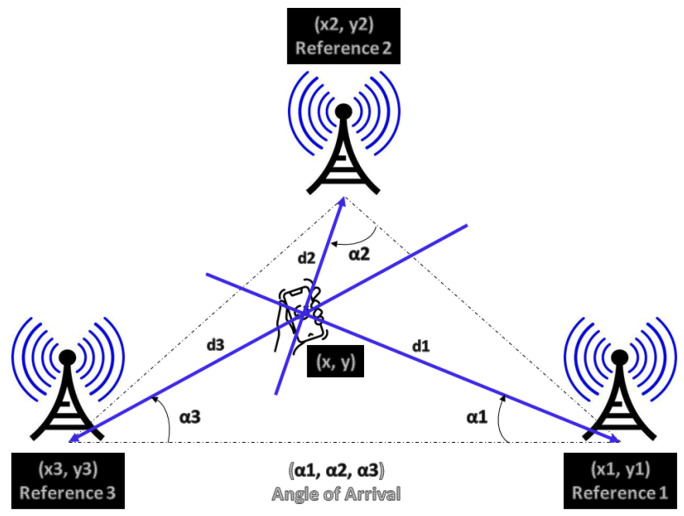
Angle of arrival method, adapted.

Using multiple receiver antennas [49], more commonly known as antenna arrays, it is possible to estimate the angle at which the transmitted signal impinges on the receivers; see Figure 1.

The AoA approach uses this angle $\alpha_{i}$ and the antenna positions $\left(x_{i},y_{i}\right)$, which are known in advance, to estimate and determine the two-dimensional (2D) (x,y) or three-dimensional (3D) (x,y,z) position of a transmitter. These data can be used for tracking or navigation purposes. Equation (2) represents the generic principle to obtain the object position by the AoA method.

(2) $$\begin{array}{cc} x & =d_{i}\times cos\left(\alpha_{i}\right)+x_{i}\\ y & =d_{i}\times sin\left(\alpha_{i}\right)+y_{i} \end{array}$$

and *i* is the antenna number 1, 2, or 3.

While the distance between the transmitter and receiver increases, the two best-known features of AoA are the accuracy deterioration of the transmitter’s estimated position and the hardware is much more expensive and complex than in other techniques.

#### 3.1.3. Time Difference of Arrival (TDoA)

This method measures the difference in TOA at two or more different sensors, in other words, it exploits the relative position of a mobile transmitter based on the different signal propagation times of the transmitter and the multiple receivers. To calculate the perfect location of a transmitter is required, at least, three receivers and a strict synchronization between them [50]. Unlike ToF techniques where synchronization is needed between the transmitter and the receiver, in TDoA, only synchronization between the receivers is required. The signal bandwidth, the sampling rate, and a nondirect line of sight between the transmitter and the receivers will affect the accuracy of the system.

### 3.2. Localization Methods

Indoor localization refers to the use of methods to determine the location of a device or person inside a building or other enclosed structure. Several different technologies can be used for indoor localization. So in this subsection, will be presented two high-tech and industrial technologies: ultra-wideband (UWB), and ultrasound (US).

#### 3.2.1. Ultra-Wideband (UWB)

The radio signals can penetrate a variety of materials, although metals and liquids can interfere with it. So, this immunity to interference from other signals makes the ultra-wideband very attractive for indoor localization [51]. This radio technology can enable the very accurate measure of the ToF, leading to centimeter accuracy distance/location measurement. This system features two methods: passive and active. The first one does not use a UWB tag and takes advantage only of the signal reflection to obtain the object or person’s position. In this specific case, it is necessary to know, in advance, where the system transmitters and receivers are located, to later be able to calculate where the object or person is, through its intersection in the signals sent and received between transmitters and receivers. On the other hand, an active UWB-based positioning system makes use of a battery-powered UWB tag. In this case, the system locates and tracks the tag, in indoor environments, by transmitting ultra-short UWB pulses from it to the fixed UWB sensors. The sensors send the collected data, via a wireless network, to the software platform, which then analyses, computes, and displays the position of the UWB tag in real-time. Furthermore, the application of UWB in indoor environments has the advantages of long battery life for UWB tags, robust flexibility, high data rates, high penetrating power, low power consumption and transmission, good positioning accuracy and performance, and little or no interference and multipath effects. In addition, UWB is expensive to scale because of the need to deploy more UWB sensors in a wide coverage area to improve performance.

#### 3.2.2. Ultrasound (US)

Mostly supported by the ToF technique, US localization technology [52] calculates the distance between tags and nodes using sound velocity and ultrasound signals. Though the sound velocity can vary with atmospheric or weather conditions, factors such as humidity and temperature affect its propagation. However, the implementation of specific filter algorithms, based on complex signal processing, can reduce the environmental noise and consequently increase the localization accuracy. To provide system synchronization, usually, the ultrasound signal is supplemented by radio frequency (RF) pulses.

## 4. Methodology

This section serves as a comprehensive guide to the research design, data collection methods, and analytical techniques utilized to ensure the validity, reliability, and robustness of paper findings. By transparently outlining the steps taken to gather and analyze data, this section is structured to provide a clear understanding of the research process, allowing for a critical assessment of the study’s methodology and its implications for the interpretation of results. Section 4.1, named Testing Scenario and Indoor Localization Systems, presents the industrial indoor scenario where all the tests were developed and the three used indoor localization systems. Section 4.2, called Data Acquisition, presents the original data acquired from each localization system, and the last, called Data Transformation or Section 4.3, addresses the conversion of the points obtained in the various indoor systems to the robot’s referential.

### 4.1. Testing Scenario and Indoor Localization Systems

**Figure 2:**
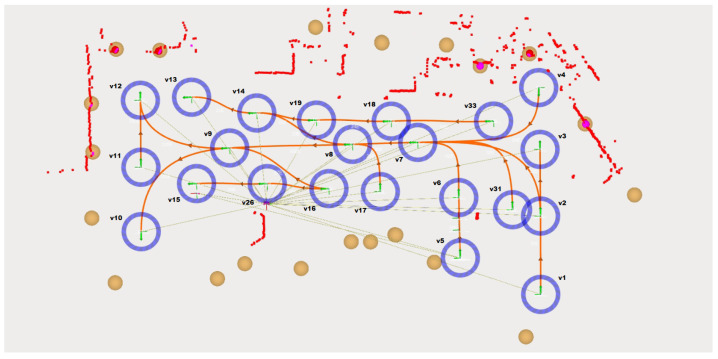
Test scene. Image exported from Robot Operating System Visualization (RVIZ).

Nowadays, in mobile robotics, the map is given by natural markers/contours of the environment, however, for these tests, beacons with a high reflection rate were used, represented by the brown circles in Figure 2. They assume always the same position, on the factory floor, so this allows the mobile platform localization system to compare, in real time, the previous 2D beacon location, saved in a file, and the live position, which is given by the reflection of the security laser waves.

In this case, the robot localization system only gives relevance to the beacon position and odometry to estimate its position and orientation. The robot localization system needs to see at least two beacons, represented by red circles inside the beacons circles, to determine the exact robot location, with this only giving relevance to odometry.

The trajectory is composed of waypoints/vertices, blue circles, and edges, which are assumed to be the connecting paths between the vertices and through which the autonomous mobile robot moves, the orange splines. All these edges are bidirectional, so the AMR can move to both sides. Both vertices and edges are associated with a specific ID number, which is randomly assigned by the trajectory editor module, giving only relevance to the fact that each vertex and each edge has a unique identification number.

Indoor localization systems can be used for a variety of purposes, such as improving navigation, providing location-based services, and tracking the movements of people or objects within a building.

Several factors can affect the accuracy and reliability of indoor localization systems, including the type of technology used, the layout and environment of the building, and the accuracy of the underlying maps or reference points. To achieve reliable and accurate indoor localization, it is often necessary to use a combination of different technologies and techniques and to carefully calibrate and maintain the system.

Overall, indoor localization systems are an important tool for improving the efficiency, safety, and experience of people inside buildings, and have many potential applications in a wide range of industries.

The next subsections introduce and attend to the three industrial localization systems (Qorvo, Eliko Kio, and Marvelmind) used for the comparison announced by this paper. One last indoor localization system will be presented, the extended Kalman filter (EKF) beacons, which is considered the test’s ground truth.

#### 4.1.1. Qorvo

**Figure 3:**
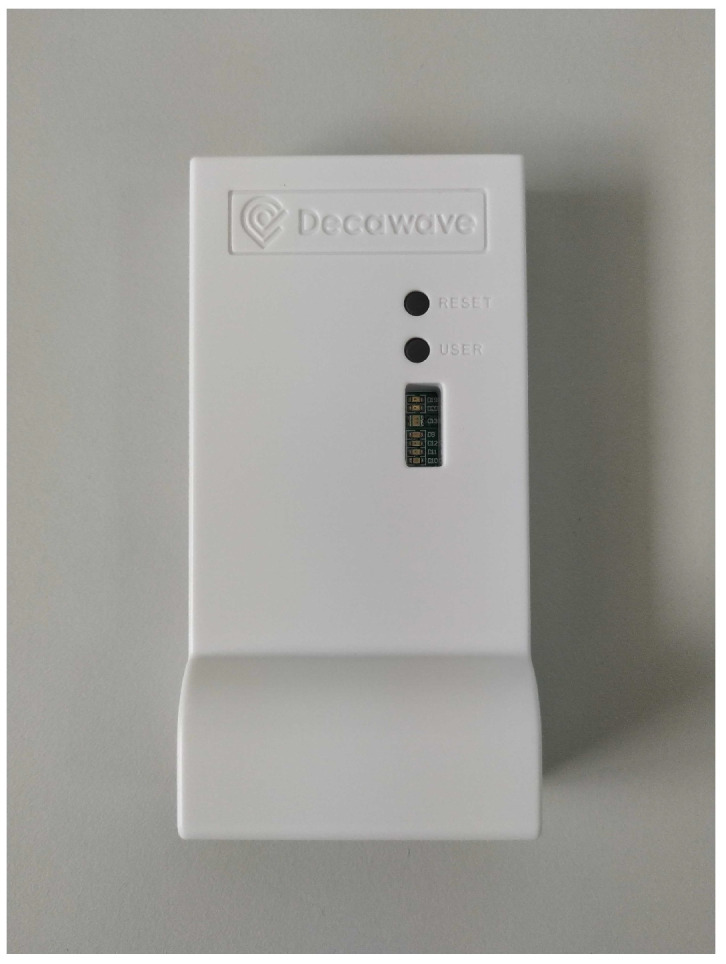
Qorvo tag.

Qorvo’s ultra-wideband technology [53], supported by Decawave’s Impulse Radio, allows for the location of tags in indoor environments (Figure 3), with high precision and at a very low cost compared to other solutions on the market, such as Pozyx [54]. Other main features of this system are secure low-power and low-latency data communication.

#### 4.1.2. Eliko Kio

**Figure 4:**
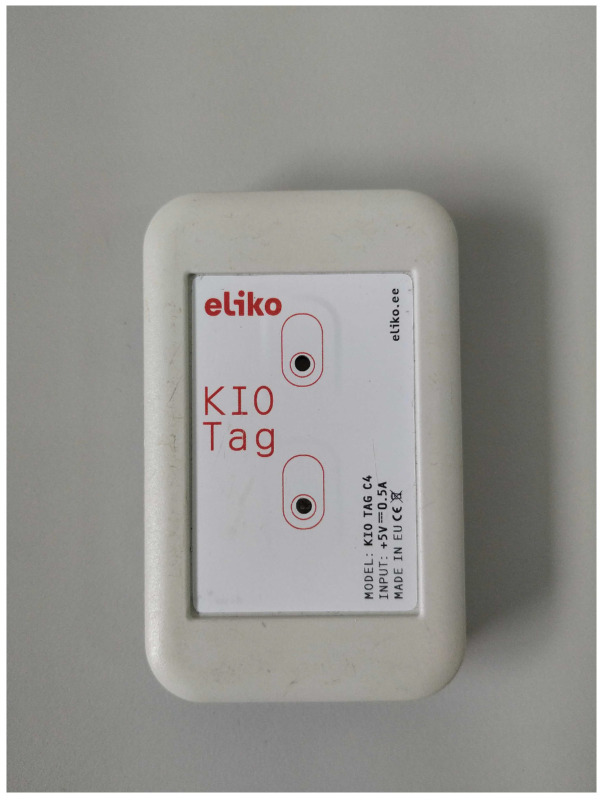
Eliko KIO tag.

The KIO system, developed by Eliko [55], is intended for 2D/3D indoor positioning of mobile UWB tags (Figure 4) in relation to fixed position UWB anchors. Based on the time of flight measurements of radio pulses traveling between tags and anchors, the 2D location consists of at least three anchors and one mobile tag. With regard to the 3D location, the KIO system needs one more anchor. Due to the low intensity of emitted radio signals, KIO devices could be used for human tracking, but the positioning frequency decreases when the number of active tags increases.

#### 4.1.3. Marvelmind

**Figure 5:**
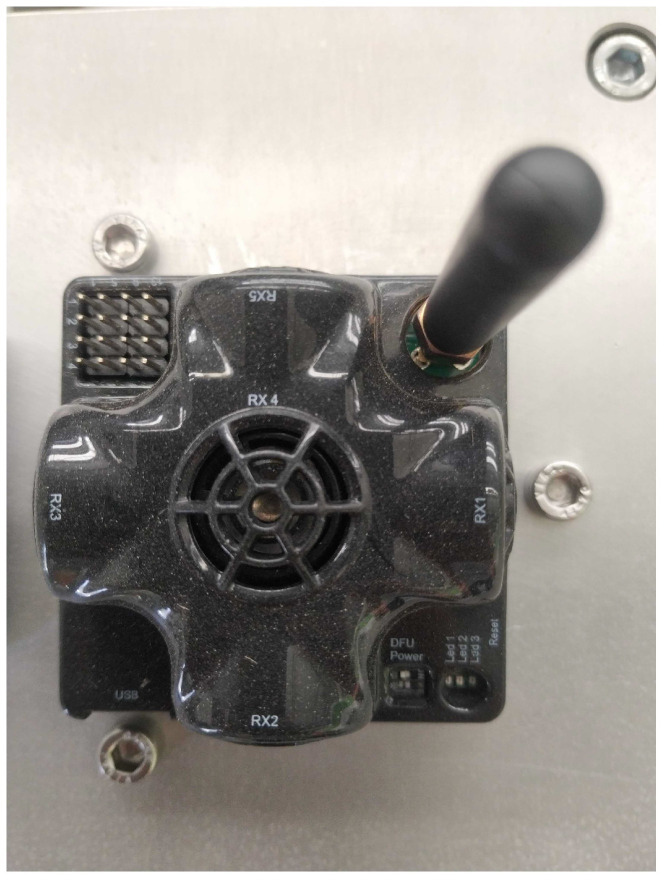
Marvelmind tag.

The indoor positioning system by Marvelmind robotics [56], Figure 5, uses ultrasound ranging to find the position of one or more mobile sensor modules, also known as hedgehogs. Ultrasound ranging is also used by beacons, the transmitters, to determine their relative position. Therefore, the Marvelmind system is self-calibrating and the sensor modules have built-in rechargeable batteries. By the application programming interface (API), it is possible to choose if a module is a beacon or a hedgehog, which allows for greater system flexibility. The maximum update rate for tracking a single hedgehog is 16 Hz. However, in addition to ultrasonics, Marvelmind may also incorporate other communication technologies for data transmission and communication between beacons and tracked objects. Bluetooth and radio frequency communication are commonly used in conjunction with ultrasonics to enhance the capabilities of indoor positioning systems.

#### 4.1.4. EKF Beacons—Ground Truth

Table 1 shows a small comparison between the different systems. All the data were taken from their datasheets.

**Figure 6:**
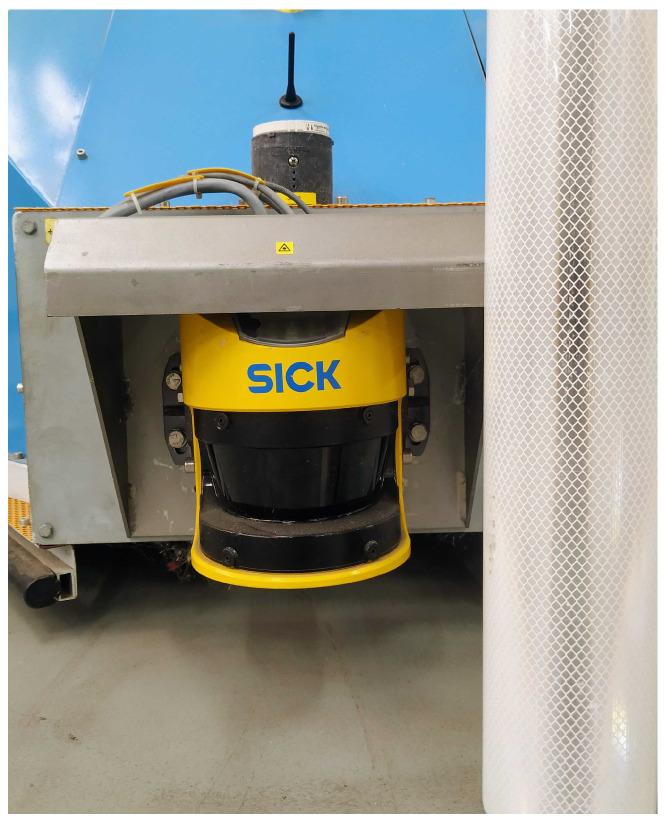
Beacon example.

The high intensity/reflection of these beacons (Figure 6) and the large number of samples present on the factory floor gives the robot localization system excellent accuracy and repeatability, making it the ground truth of these tests. So, in Table 2, it is possible to see the comparison between the robot position, given by its localization system, in each vertex, and the vertex position values presented in the trajectory data file. All these values were taken based on the robot map referential, which typically refers to the coordinate system or frame of reference used by a robot to represent and navigate within its environment. This referential is crucial for the robot to understand its position, orientation, and movement relative to the surrounding space.

Regarding the integration of indoor localization systems, in industry, has the potential to improve productivity, efficiency, and safety, as well as to create new opportunities for innovation and value creation. Therefore, the next subsection exhibited the implementation of the different systems listed before, either on the AMR or on the industrial scenario.

#### 4.1.5. Industrial Scenario—Systems Integration

To cover the whole robot map area with the different indoor localization systems, some preliminary tests were carried out that allowed us to conclude the data present in Table 3.

**Figure 7:**
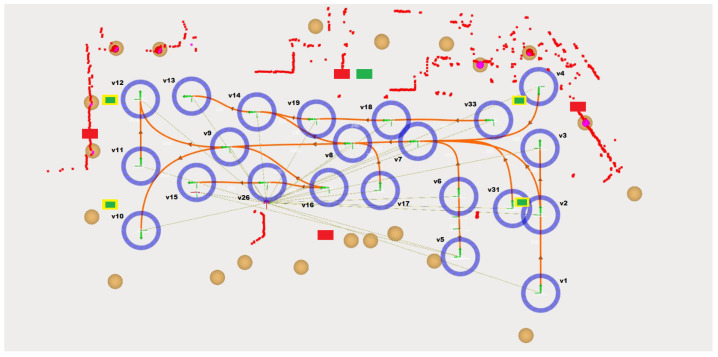
Sensors distribution in the industrial environment.

Figure 7, supported by Figure 2, highlights the distribution of the different localization systems across the plant floor, the colored rectangles distributed in the image, as well as the robot trajectories and the beacon map. A trajectory that spans the entire range of action of the different indoor localization systems was scaled to best evaluate them, because the greater the distance of the robot to them, the greater the error associated with the robot’s position.

In the previous figure, the Eliko Kio system is illustrated by the four red rectangles, two of them at the center of the image and the others on each side. Regarding the Marvelmind system, it was possible to cover all the scene areas with only four tags, illustrated by the yellow rectangles. The only system where it was essential to use another tag was the Qorvo system, exemplified by five green rectangles, where four of them have a similar position to Marvelmind system tags. All these tags, regardless of the location system they are associated with, are both in the same referential.

#### 4.1.6. Autonomous Mobile Robot—Systems Integration

**Figure 8:**
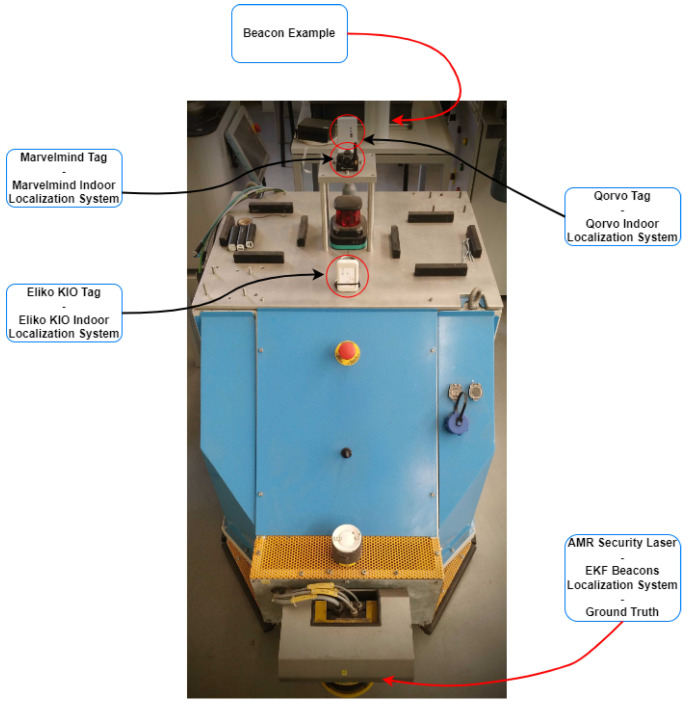
Mobile platform sensors integration—system’s architecture.

On the mobile platform, as it is possible to see in Figure 8, each tag has a specific position and all of them were powered by a portable power bank.

To be able to compare the positions obtained by the ground truth and the different indoor localization systems, it was necessary to match the positions of the sick laser with each of the onboard tags. In this way, Table 4 represents the transformations of beacons data, in each vertex as described in Table 2 on robot row, to the three localization systems used in this case. These conversions were based on Equations (3) and (4).

(3) $$\begin{array}{cc} X_{New} & =cos\left(Theta_{Beacons}\right)\times \vec{\Delta }X+X_{Beacons} \end{array}$$

where $X_{New}$ corresponds to the new *X* value of the new point, $X_{Beacons}$ represents the *X* value of the original point, and $\vec{\Delta }X$ is the modulus of the difference between the last two values. The last parameter of the equation, $Theta_{Beacons}$, assumes the angle, in radians, between the robot referential and the map referential in the original point.

(4) $$\begin{array}{cc} Y_{New} & =sin\left(Theta_{Beacons}\right)\times \vec{\Delta }X+Y_{Beacons} \end{array}$$

here, $Y_{New}$ corresponds to the new *Y* value of the new point, and $Y_{Beacons}$ represents the *Y* value of the original point. The last two parameters, $Theta_{Beacons}$ and $\vec{\Delta }X$, are the same exposed in the last equation because the sensors were aligned by the *X* referential.

### 4.2. Data Acquisition

This subsection exposes the robot pose received by each localization system in each vertex from the robot’s map and their correspondence to the beacon data.

#### 4.2.1. Beacons Data

Table 5 shows the average, the standard deviation, and the maximum and minimum values of the AMR localization system in each vertex.

#### 4.2.2. Marvelmind

Table 6 shows the average, the standard deviation, and the maximum and minimum values of the Marvelmind localization system in each vertex.

**Figure 9:**
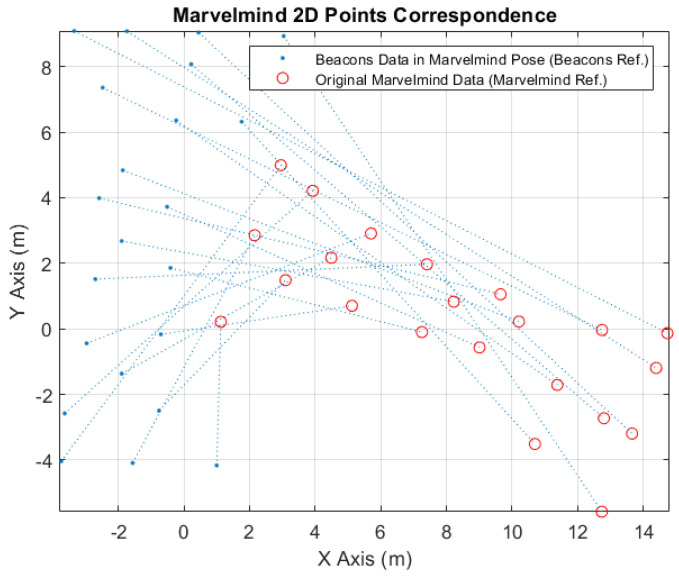
Marvelmind 2D Points Correspondence.

In Figure 9, it is possible to see the correspondence points between the converted beacons data to the Marvelmind robot tag position (blue points) and the received original Marvelmind data (red circles).

#### 4.2.3. Eliko Kio

Table 7 shows the average, the standard deviation, and the maximum and minimum values of the Eliko Kio localization system in each vertex.

**Figure 10:**
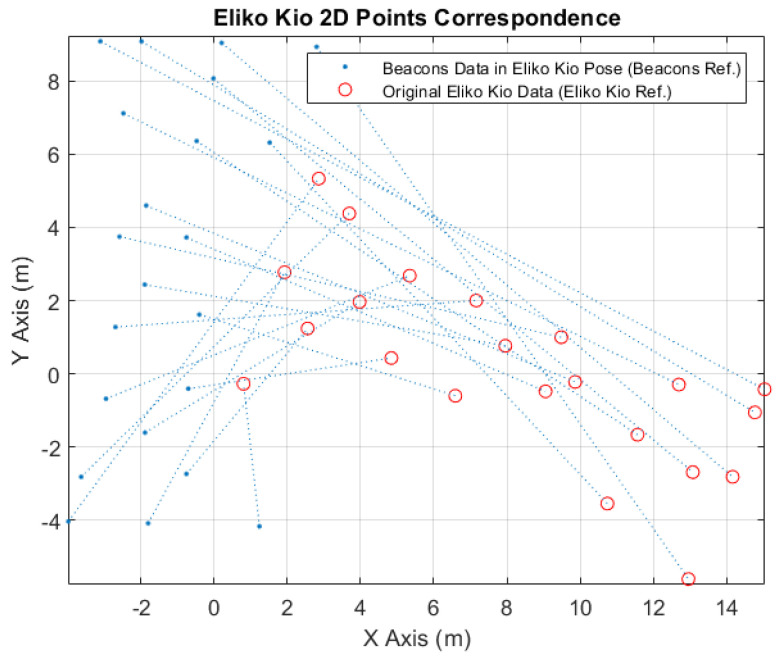
Eliko Kio 2D Points Correspondence.

In Figure 10, it is possible to see the correspondence points between the converted beacons data to the Eliko Kio robot tag position (blue points) and the received original Eliko Kio data (red circles).

#### 4.2.4. Qorvo

Table 8 shows the average, the standard deviation, and the maximum and minimum values of the Qorvo localization system in each vertex.

**Figure 11:**
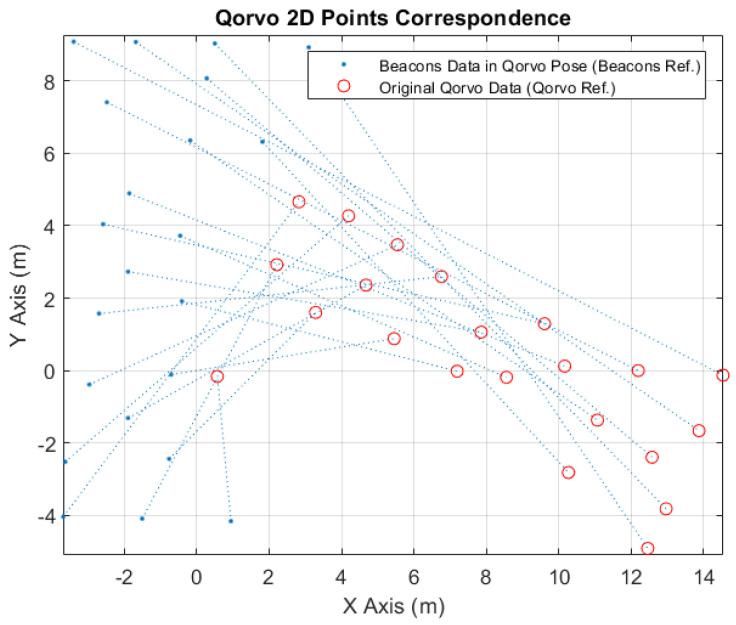
Qorvo 2D points correspondence.

In Figure 11, it is possible to see the correspondence points between the converted beacons data to the Qorvo robot tag position (blue points) and the received original Qorvo data (red circles).

### 4.3. Data Transformation

This subsection will present the transformation matrix, which concerns the conversion of the points in each location system’s referential to their coordinates in the robot’s map referential. It will also be possible to observe, through figures, the approximation of the original points of each location system to the respective values acquired by ground truth.

After acquiring the various sets of points, in the different references, the next calculation would be to calculate the respective transform between them (Marvelmind to ground truth, Eliko Kio to ground truth, and Qorvo to ground truth). Based on the least squares (LS) approximation and with the help of MatLab, the following transformation matrices and respective errors, in each of the coordinates, were obtained.

#### 4.3.1. Marvelmind

Equations (5)–(7) represent the transformation matrix from the Marvelmind referential to the Beacons referential, the ground truth referential.

(5) $$\begin{array}{c} 2D\;Translation\;Matrix=\left[\begin{array}{c} 1.6779\\ -4.9422 \end{array}\right] \end{array}$$

(6) $$\begin{array}{c} 2D\;Rotation\;Matrix=\left[\begin{array}{cc} -0.3206 & -0.9472\\ 0.9472 & -0.3206 \end{array}\right] \end{array}$$

(7) $$\begin{array}{c} Angle=252.535{}^{\circ} \end{array}$$

**Figure 12:**
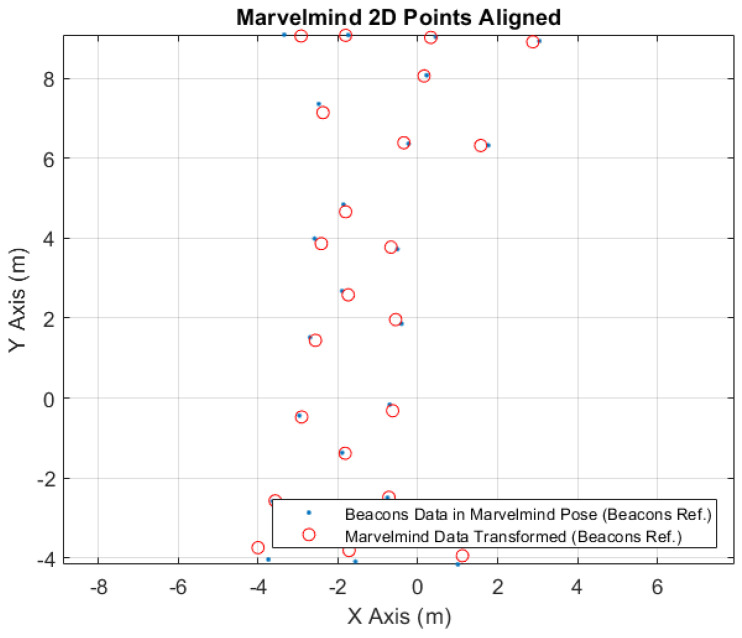
Marvelmind 2D points aligned.

In Figure 12, it is possible to see the aligned points to the Marvelmind localization system. The blue points represent the converted robot position points, acquired from the robot location system, to the Marvelmind robot tag position. The red circles are the original robot location points, acquired from the Marvelmind system, transformed to the robot location system referential; see Table 9. Comparing these to types of points, after the conversion, it is possible to obtain the coordinate errors exposed in Table 10.

#### 4.3.2. Eliko Kio

Equations (8)–(10) represent the transformation matrix from the Eliko Kio referential to the Beacons referential, the ground truth referential.

(8) $$\begin{array}{c} 2D\;Translation\;Matrix=\left[\begin{array}{c} 1.3654\\ -5.0303 \end{array}\right] \end{array}$$

(9) $$\begin{array}{c} 2D\;Rotation\;Matrix=\left[\begin{array}{cc} -0.3060 & -0.9520\\ 0.9520 & -0.3060 \end{array}\right] \end{array}$$

(10) $$\begin{array}{c} Angle=253.213{}^{\circ} \end{array}$$

**Figure 13:**
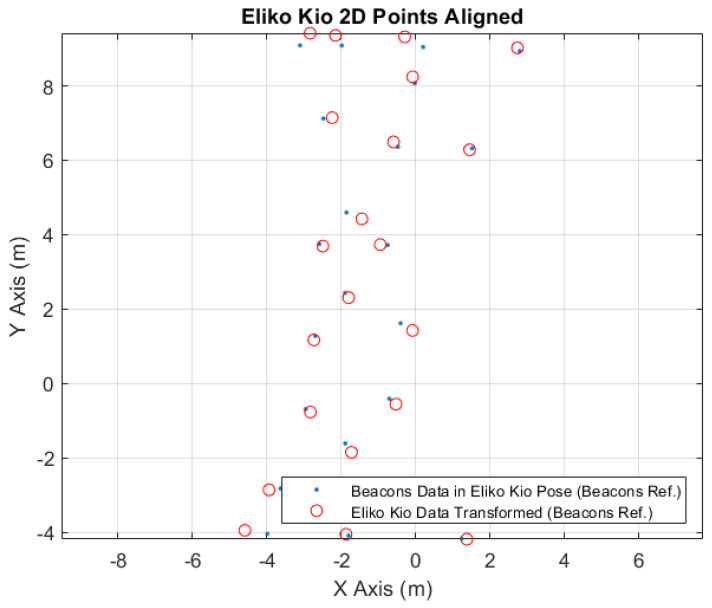
Eliko Kio 2D points aligned.

In Figure 13, it is possible to see the aligned points to the Eliko Kio localization system. The blue points represent the converted robot position points, acquired from the robot location system, to the Eliko Kio robot tag position. The red circles are the original robot location points, acquired from the Eliko Kio system, transformed to the robot location system referential; see Table 11. Comparing these to types of points, after the conversion, it is possible to obtain the coordinate errors exposed in Table 12.

#### 4.3.3. Qorvo

Equations (11)–(13) represent the transformation matrix from the Qorvo referential to the Beacons referential, the ground truth referential.

(11) $$\begin{array}{c} 2D\;Translation\;Matrix=\left[\begin{array}{c} 1.8242\\ -4.6445 \end{array}\right] \end{array}$$

(12) $$\begin{array}{c} 2D\;Rotation\;Matrix=\left[\begin{array}{cc} -0.3297 & -0.9441\\ 0.9441 & -0.3297 \end{array}\right] \end{array}$$

(13) $$\begin{array}{c} Angle=252.609{}^{\circ} \end{array}$$

**Figure 14:**
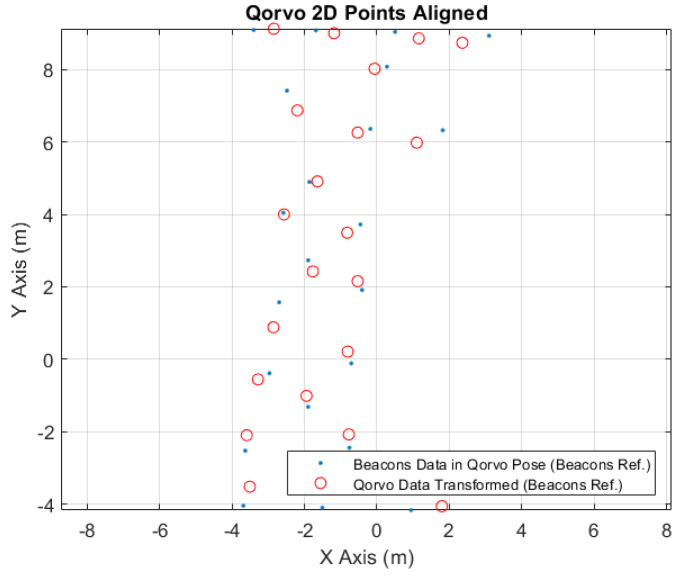
Qorvo 2D points aligned.

In Figure 14, it is possible to see the aligned points to the Qorvo localization system. The blue points represent the converted robot position points, acquired from the robot location system, to the Qorvo robot tag position. The red circles are the original robot location points, acquired from the Qorvo system, transformed to the robot location system referential; see Table 13. Comparing these to types of points, after the conversion, it is possible to obtain the coordinate errors exposed in Table 14.

## 5. Results

In the results section of this paper, the research outcomes are unveiled, providing a detailed account of the data obtained through meticulous analysis. This section serves as a culmination of the study’s investigative efforts, presenting a comprehensive depiction of the key findings about the research questions and objectives outlined earlier.

After presenting the results obtained with the different indoor localization systems and comparing the respective error values in each map point with the ground truth used (see Table 15), it can be stated that the Marvelmind system contains less error than Eliko Kio and Qorvo systems, being, in this environment and study scenario, the most accurate and precise module.

**Figure 15:**
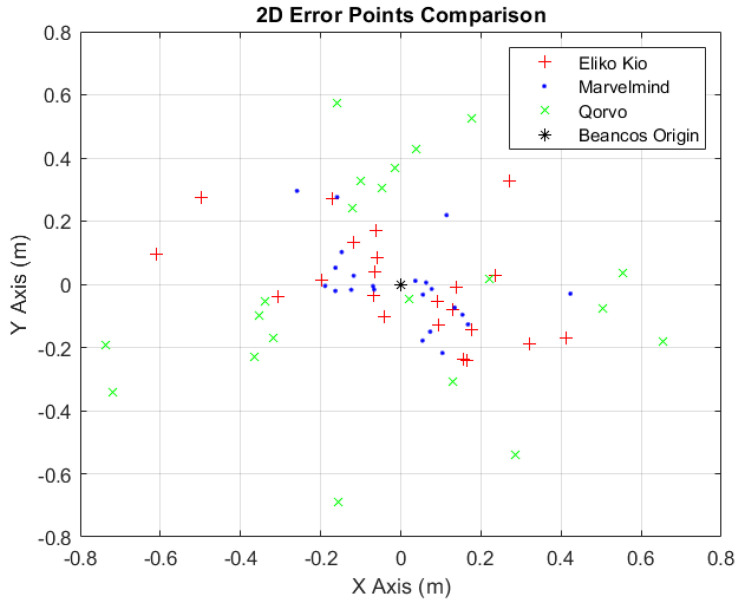
Two-dimensional error points comparison.

The blue dots, in Figure 15, refer to the Marvelmind indoor localization system. They are the closest to the point of origin (0,0), presented by the symbol ∗, alluding to ground truth values, thus assuming that it is the best-tested indoor localization system. The + symbols, in red, refer to the Eliko KIO localization system, and the *x* symbols refer to the Qorvo localization system.

**Figure 16:**
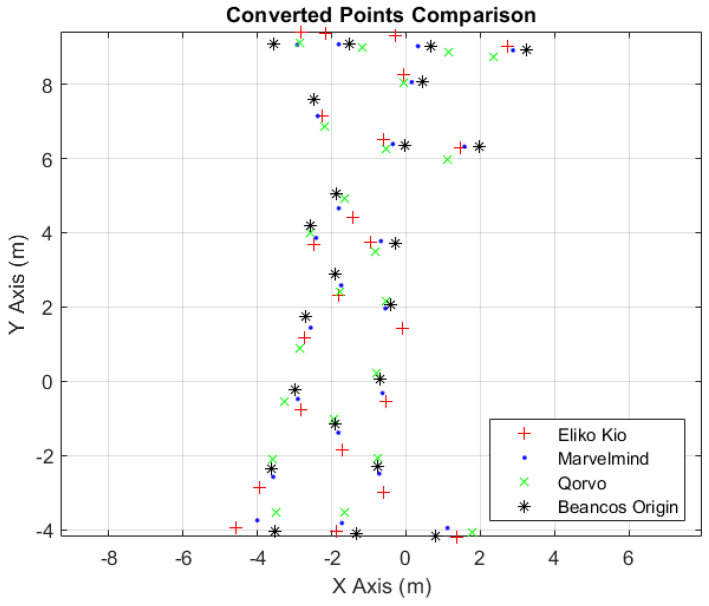
Two-dimensional converted points comparison.

A more detailed and illustrative analysis is presented in Figure 16, illustrating more intuitively the difference, in terms of distance allusive to ground truth values, of the three used systems at each of the vertices.

Table 16 presents the best indoor localization system, in each vertex, which is highlighted with the respective color, according to the image caption in Figure 16. The blue color, alluding to Marvelmind, is the most repeated throughout the table, confirming it as the best system of the three selected.

However, it is always necessary to take into account that there are always areas of the map where the different systems have difficulty in having precision, and even accuracy, in locating the AMR, leading to the so-called outliers. These critical points have to do with the positioning or distribution of the different modules, the various location systems, as well as the proximity of the AMR to industrial machinery and surrounding structures of the scenario itself, consisting mostly of iron, eventually influencing the signal propagation and the radio wave reflections.

In summary, there are several approaches to indoor localization for autonomous mobile robots and other different mobile platforms, each with its own set of advantages and disadvantages. The best approach for a given application will depend on the specific requirements and constraints of the environment and the accuracy can vary depending on the specific technologies and infrastructure used. After this analysis, it is possible to conclude that the Marvelmind system is the most accurate and the one that can cover a larger working area with the least number of tags. However, it is the most expensive system of all presented. As far as our case study is concerned, that is, for real-time detection of mobile platforms, such as AMRs, forklifts, or even logistics trains in industrial environments, any of the three systems will work, because they all have satisfactory results with errors below half a meter, which will always allow for safe, accurate, and optimized path planning for all AMRs.

## 6. Conclusions

This article tests three industrial indoor localization solutions: Qorvo, Eliko Kio, and Marvelmind, supported by two indoor localization methods: ultra-wideband (Qorvo and Eliko Kio) and Uultrasound (Marvelmind). A multicomparison between these three different indoor localization systems and a robot localization system (ground truth) was proposed. To optimize the data obtained by each system, the data acquired by the AMR location system were previously transformed to the position of each of the tags integrated on top of the AMR. Finally, an approximation was used, through MATLAB, using the method of least squares, of the points obtained by each localization system to the respective. It was possible to conclude that the Marvelmind system is the most accurate, but, for our proposal, any of the other systems could be used, because they all have errors below half a meter, which will always allow for safe, accurate, and optimized path planning for all AMRs when planning the paths for the different robots, taking into account the position of the different mobile platforms on the factory floor that are not managed by the robot fleet manager (forklifts, logistics trains).

A seminal contribution of this work lies in its comprehensive examination of three industrial localization systems in real time, coupled with a sophisticated and in-depth analysis. By systematically comparing and contrasting these disparate systems, this study endeavors to unearth nuanced insights into their respective functionalities. The intricate examination of real-time industrial localization not only elucidates the dynamic landscape of these technologies but also underscores their practical implications and potential advancements. This analytical approach offers a multifaceted perspective, fostering a deeper understanding of the intricate interplay between diverse industrial localization systems and providing a foundation for informed decision making in the realm of contemporary technological applications. Various quantitative and qualitative results of the different systems were presented, which could help readers make a future choice when purchasing an indoor localization system on the market.

As future work, it will be interesting to validate one of these indoor localization systems integrated into different forklifts or logistic trains and interaction with the TEA* Algorithm, the AMRs path planning algorithm, in real time and in a real environment.

## Figures and Tables

**Table 1:** Device comparison—some features.

Localization System	Precision	Ease of Deployment	Power Consumption	Scalability	Environmental Considerations
Qorvo	±10 cm	yes	low power sleep mode: 15 μA	easy	−40 °C…+85 °C
Eliko Kio	±15 cm	yes	155 mA in Rx mode 95 mA in Tx mode	easy	−20…+55 °C (USB powered device)
Marvelmind	±2 cm	yes	900–1000 mAh 3.6 V	easy	−40 °C…+50 °C

**Table 2:** Ground truth selection process.

Vertex ID	5			6			10		
Pose	X	Y	Theta	X	Y	Theta	X	Y	Theta
Map	1.973	6.334	−3.140	−0.017	6.347	3.131	0.783	−4.156	−0.025
Robot	1.977	6.324	−3.131	−0.019	6.357	3.132	0.786	−4.159	−0.023
Diff.	0.004	0.010	0.008	0.002	0.009	0.001	0.003	0.003	0.002
Vertex ID	12			11			15		
Pose	X	Y	Theta	X	Y	Theta	X	Y	Theta
Map	−3.548	−4.050	3.127	−1.341	−4.100	3.120	−0.746	−2.282	−1.566
Robot	−3.525	−4.043	3.127	−1.346	−4.095	3.122	−0.763	−2.281	−1.553
Diff.	0.024	0.007	0.001	0.005	0.005	0.002	0.017	0.001	0.014
Vertex ID	26			16			9		
Pose	X	Y	Theta	X	Y	Theta	X	Y	Theta
Map	−0.672	0.048	−1.566	−0.445	2.076	−1.566	−1.885	−1.155	−1.545
Robot	−0.706	0.047	−1.565	−0.414	2.074	−1.542	−1.903	−1.153	−1.534
Diff.	0.033	0.001	0.001	0.031	0.002	0.024	0.019	0.001	0.011
Vertex ID	33			18			19		
Pose	X	Y	Theta	X	Y	Theta	X	Y	Theta
Map	−2.504	7.572	−1.566	−2.605	4.198	−1.566	−2.717	1.734	−1.566
Robot	−2.482	7.573	−1.556	−2.586	4.204	−1.564	−2.702	1.732	−1.551
Diff.	0.021	0.001	0.011	0.019	0.005	0.002	0.014	0.002	0.015
Vertex ID	14			13			17		
Pose	X	Y	Theta	X	Y	Theta	X	Y	Theta
Map	−3.011	−0.226	−1.566	−3.597	−2.359	−1.566	−0.294	3.765	3.131
Robot	−2.974	−0.227	−1.522	−3.635	−2.363	−1.561	−0.295	3.721	3.136
Diff.	0.036	0.002	0.044	0.038	0.004	0.006	0.001	0.044	0.005
Vertex ID	8			4			3		
Pose	X	Y	Theta	X	Y	Theta	X	Y	Theta
Map	−1.879	2.897	−1.566	−3.561	9.092	−0.023	−1.524	9.073	3.120
Robot	−1.900	2.893	−1.553	−3.560	9.087	0.001	−1.524	9.076	3.123
Diff.	0.021	0.004	0.014	0.001	0.005	0.024	0.000	0.004	0.004
Vertex ID	2			1			31		
Pose	X	Y	Theta	X	Y	Theta	X	Y	Theta
Map	0.674	9.022	3.120	3.257	8.948	3.114	0.438	8.106	3.131
Robot	0.662	9.033	3.120	3.256	8.927	3.121	0.438	8.076	−3.132
Diff.	0.012	0.012	0.001	0.001	0.021	0.007	0.000	0.030	6.263
Vertex ID	7								
Pose	X	Y	Theta						
Map	−1.879	5.051	−1.578						
Robot	−1.865	5.053	−1.549						
Diff.	0.013	0.002	0.030						

**Table 3:** Minimum number of tags per localization system.

Localization System	Minimum Tags/Beacons Number	Detection Type
Qorvo	5	Tags
Eliko Kio	4	Tags
Marvelmind	4	Tags
EKF Beacons (AMR)	2	Beacons

**Table 4:** Beacons data conversion.

Localization Systems	Vertex ID	5		6		10	
	Delta X	X	Y	X	Y	X	Y
Marvelmind	0.215	1.762	6.322	−0.234	6.359	1.001	−4.164
Qorvo	0.16	1.817	6.322	−0.179	6.358	0.946	−4.163
Eliko Kio	0.455	1.522	6.319	−0.474	6.361	1.241	−4.170
Localization Systems	Vertex ID	12		11		15	
	Delta X	X	Y	X	Y	X	Y
Marvelmind	0.215	−3.740	−4.040	−1.561	−4.091	−0.759	−2.496
Qorvo	0.16	−3.685	−4.040	−1.506	−4.092	−0.760	−2.441
Eliko Kio	0.455	−3.980	−4.036	−1.801	−4.086	−0.754	−2.736
Localization Systems	Vertex ID	26		16		9	
	Delta X	X	Y	X	Y	X	Y
Marvelmind	0.215	−0.704	−0.168	−0.408	1.859	−1.895	−1.368
Qorvo	0.16	−0.705	−0.113	−0.409	1.914	−1.897	−1.313
Eliko Kio	0.455	−0.703	−0.408	−0.401	1.619	−1.887	−1.608
Localization Systems	Vertex ID	33		18		19	
	Delta X	X	Y	X	Y	X	Y
Marvelmind	0.215	−2.479	7.358	−2.584	3.989	−2.698	1.517
Qorvo	0.16	−2.480	7.413	−2.585	4.044	−2.699	1.572
Eliko Kio	0.455	−2.476	7.118	−2.583	3.749	−2.693	1.277
Localization Systems	Vertex ID	14		13		17	
	Delta X	X	Y	X	Y	X	Y
Marvelmind	0.215	−2.964	−0.442	−3.633	−2.578	−0.510	3.722
Qorvo	0.16	−2.967	−0.387	−3.634	−2.523	−0.455	3.722
Eliko Kio	0.455	−2.952	−0.682	−3.631	−2.818	−0.750	3.724
Localization Systems	Vertex ID	8		4		3	
	Delta X	X	Y	X	Y	X	Y
Marvelmind	0.215	−1.896	2.678	−3.345	9.087	−1.739	9.080
Qorvo	0.16	−1.897	2.733	−3.400	9.087	−1.684	9.079
Eliko Kio	0.455	−1.892	2.438	−3.105	9.087	−1.979	9.085
Localization Systems	Vertex ID	2		1		31	
	Delta X	X	Y	X	Y	X	Y
Marvelmind	0.215	0.447	9.038	3.041	8.931	0.223	8.074
Qorvo	0.16	0.502	9.037	3.096	8.930	0.278	8.075
Eliko Kio	0.455	0.207	9.043	2.801	8.936	−0.017	8.072
Localization Systems	Vertex ID	7					
	Delta X	X	Y				
Marvelmind	0.215	−1.861	4.838				
Qorvo	0.16	−1.862	4.893				
Eliko Kio	0.455	−1.855	4.598				

**Table 5:** AMR localization system—Beacons data.

Vertex ID	5			6			10		
Data	X	Y	Theta	X	Y	Theta	X	Y	Theta
AVG	1.977	6.324	−3.131	−0.019	6.357	3.132	0.786	−4.159	−0.023
Std. Deviation	0.001	0.001	0.000	0.002	0.001	0.000	0.002	0.001	0.000
Max	1.979	6.327	−3.131	−0.014	6.358	3.132	0.788	−4.157	−0.023
Min	1.975	6.323	−3.131	−0.021	6.355	3.131	0.782	−4.160	−0.024
Diff.	0.003	0.004	0.000	0.007	0.003	0.001	0.006	0.004	0.001
Vertex ID	12			11			15		
Data	X	Y	Theta	X	Y	Theta	X	Y	Theta
AVG	−3.525	−4.043	3.127	−1.346	−4.095	3.122	−0.763	−2.281	−1.553
Std. Deviation	0.001	0.001	0.000	0.001	0.000	0.000	0.001	0.002	0.000
Max	−3.522	−4.042	3.127	−1.345	−4.094	3.122	−0.758	−2.274	−1.553
Min	−3.526	−4.044	3.126	−1.349	−4.095	3.122	−0.764	−2.283	−1.553
Diff.	0.004	0.002	0.002	0.004	0.001	0.000	0.006	0.009	0.001
Vertex ID	26			16			9		
Data	X	Y	Theta	X	Y	Theta	X	Y	Theta
AVG	−0.706	0.047	−1.565	−0.414	2.074	−1.542	−1.903	−1.153	−1.534
Std. Deviation	0.001	0.001	0.000	0.004	0.001	0.000	0.002	0.001	0.000
Max	−0.703	0.050	−1.565	−0.409	2.074	−1.541	−1.899	−1.148	−1.534
Min	−0.706	0.045	−1.565	−0.425	2.072	−1.544	−1.905	−1.155	−1.535
Diff.	0.003	0.005	0.001	0.017	0.003	0.002	0.006	0.007	0.001
Vertex ID	33			18			19		
Data	X	Y	Theta	X	Y	Theta	X	Y	Theta
AVG	−2.482	7.573	−1.556	−2.586	4.204	−1.564	−2.702	1.732	−1.551
Std. Deviation	0.003	0.002	0.000	0.003	0.002	0.000	0.001	0.002	0.000
Max	−2.479	7.575	−1.555	−2.582	4.206	−1.563	−2.700	1.734	−1.551
Min	−2.490	7.565	−1.557	−2.594	4.196	−1.564	−2.706	1.725	−1.552
Diff.	0.011	0.010	0.001	0.012	0.010	0.001	0.005	0.009	0.001
Vertex ID	14			13			17		
Data	X	Y	Theta	X	Y	Theta	X	Y	Theta
AVG	−2.974	−0.227	−1.522	−3.635	−2.363	−1.561	−0.295	3.721	3.136
Std. Deviation	0.002	0.001	0.000	0.002	0.003	0.001	0.002	0.000	0.000
Max	−2.973	−0.226	−1.522	−3.630	−2.352	−1.560	−0.293	3.721	3.136
Min	−2.979	−0.231	−1.523	−3.638	−2.366	−1.564	−0.301	3.720	3.136
Diff.	0.007	0.005	0.000	0.008	0.014	0.004	0.008	0.002	0.000
Vertex ID	8			4			3		
Data	X	Y	Theta	X	Y	Theta	X	Y	Theta
AVG	−1.900	2.893	−1.553	−3.560	9.087	0.001	−1.524	9.076	3.123
Std. Deviation	0.001	0.002	0.000	0.001	0.004	0.000	0.001	0.001	0.000
Max	−1.897	2.898	−1.552	−3.557	9.091	0.001	−1.520	9.078	3.124
Min	−1.902	2.891	−1.553	−3.561	9.075	0.001	−1.525	9.075	3.123
Diff.	0.005	0.007	0.000	0.003	0.016	0.001	0.005	0.002	0.000
Vertex ID	2			1			31		
Data	X	Y	Theta	X	Y	Theta	X	Y	Theta
AVG	0.662	9.033	3.120	3.256	8.927	3.121	0.438	8.076	−3.132
Std. Deviation	0.003	0.001	0.000	0.002	0.005	0.001	0.002	0.001	0.000
Max	0.672	9.035	3.121	3.258	8.939	3.122	0.440	8.081	−3.131
Min	0.659	9.030	3.120	3.251	8.921	3.120	0.432	8.075	−3.132
Diff.	0.013	0.004	0.001	0.008	0.017	0.002	0.008	0.006	0.001
Vertex ID	7								
Data	X	Y	Theta						
AVG	−1.865	5.053	−1.549						
Std. Deviation	0.001	0.001	0.000						
Max	−1.864	5.056	−1.548						
Min	−1.866	5.052	−1.549						
Diff.	0.003	0.004	0.000						

**Table 6:** Indoor localization system—Marvelmind data.

Vertex ID	5			6			10		
Data	X	Y	Z	X	Y	Z	X	Y	Z
AVG	10.698	−3.511	2.540	11.381	−1.710	2.572	1.125	0.213	2.609
Std. Deviation	0.004	0.005	0.014	0.016	0.006	0.018	0.023	0.068	0.020
Max	10.712	−3.506	2.548	11.471	−1.702	2.667	1.160	0.310	2.690
Min	10.697	−3.520	2.538	11.360	−1.744	2.547	1.090	0.140	2.580
Diff.	0.015	0.014	0.01	0.111	0.042	0.120	0.070	0.170	0.110
Vertex ID	12			11			15		
Data	X	Y	Z	X	Y	Z	X	Y	Z
AVG	2.955	4.993	2.659	2.157	2.857	2.643	3.097	1.486	2.543
Std. Deviation	0.004	0.010	0.002	0.001	0.002	0.001	0.002	0.005	0.004
Max	2.968	5.019	2.663	2.160	2.863	2.646	3.103	1.516	2.548
Min	2.948	4.980	2.654	2.155	2.852	2.641	3.093	1.481	2.525
Diff.	0.020	0.039	0.009	0.005	0.011	0.005	0.01	0.035	0.023
Vertex ID	26			16			9		
Data	X	Y	Z	X	Y	Z	X	Y	Z
AVG	5.120	0.704	2.605	7.255	−0.098	2.472	4.492	2.170	2.515
Std. Deviation	0.003	0.001	0.005	0.028	0.062	0.017	0.013	0.006	0.014
Max	5.120	0.706	2.612	7.268	−0.086	2.475	4.543	2.192	2.581
Min	5.117	0.703	2.602	7.202	−0.108	2.469	4.446	2.159	2.493
Diff.	0.003	0.003	0.01	0.066	0.022	0.006	0.097	0.033	0.088
Vertex ID	33			18			19		
Data	X	Y	Z	X	Y	Z	X	Y	Z
AVG	12.744	−0.035	2.580	9.652	1.055	2.450	7.408	1.970	2.421
Std. Deviation	0.004	0.003	0.004	0.001	0.018	0.010	0.005	0.017	0.011
Max	12.757	−0.031	2.591	9.654	1.132	2.466	7.444	2.066	2.454
Min	12.739	−0.042	2.575	9.649	1.035	2.405	7.405	1.960	2.353
Diff.	0.018	0.011	0.016	0.005	0.097	0.061	0.039	0.106	0.101
Vertex ID	14			13			17		
Data	X	Y	Z	X	Y	Z	X	Y	Z
AVG	5.702	2.912	2.524	3.927	4.211	2.618	9.010	−0.568	2.412
Std. Deviation	0.005	0.013	0.003	0.003	0.004	0.002	0.015	0.007	0.035
Max	5.718	2.951	2.530	3.938	4.224	2.625	9.057	−0.561	2.518
Min	5.688	2.877	2.515	3.919	4.204	2.611	9.001	−0.601	2.382
Diff.	0.03	0.074	0.015	0.019	0.02	0.014	0.056	0.04	0.136
Vertex ID	8			4			3		
Data	X	Y	Z	X	Y	Z	X	Y	Z
AVG	8.223	0.827	2.402	14.735	−0.132	2.777	14.394	−1.192	2.767
Std. Deviation	0.001	0.002	0.004	0.001	0.004	0.001	0.020	0.048	0.011
Max	8.226	0.832	2.425	14.735	−0.132	2.777	14.504	−1.164	2.848
Min	8.216	0.821	2.393	14.733	−0.143	2.775	14.364	−1.528	2.760
Diff.	0.01	0.011	0.032	0.002	0.011	0.002	0.14	0.364	0.088
Vertex ID	2			1			31		
Data	X	Y	Z	X	Y	Z	X	Y	Z
AVG	13.660	−3.194	2.739	12.736	−5.578	2.805	12.801	−2.727	2.685
Std. Deviation	0.017	0.016	0.003	0.045	0.017	0.004	0.028	0.011	0.017
Max	13.676	−3.183	2.752	12.873	−5.563	2.810	12.826	−2.683	2.698
Min	13.565	−3.284	2.734	12.696	−5.631	2.792	12.678	−2.740	2.615
Diff.	0.111	0.101	0.018	0.177	0.068	0.018	0.148	0.057	0.083
Vertex ID	7								
Data	X	Y	Z						
AVG	10.212	0.222	2.437						
Std. Deviation	0.001	0.002	0.002						
Max	10.213	0.228	2.442						
Min	10.210	0.217	2.433						
Diff.	0.003	0.011	0.009						

**Table 7:** Indoor localization system—Eliko Kio data.

Vertex ID	5		6		10	
Data	X	Y	X	Y	X	Y
AVG	10.743	−3.547	11.569	−1.664	0.805	−0.273
Std. Deviation	0.036	0.025	0.028	0.042	0.049	0.014
Max	10.820	−3.500	11.620	−1.590	0.870	−0.240
Min	10.630	−3.630	11.530	−1.740	0.630	−0.300
Diff.	0.19	0.13	0.09	0.15	0.24	0.06
Vertex ID	12		11		15	
Data	X	Y	X	Y	X	Y
AVG	2.859	5.335	1.925	2.776	2.557	1.239
Std. Deviation	0.016	0.010	0.013	0.028	0.015	0.021
Max	2.880	5.360	1.960	2.860	2.590	1.270
Min	2.820	5.310	1.890	2.740	2.520	1.170
Diff.	0.06	0.05	0.07	0.12	0.07	0.1
Vertex ID	26		16		9	
Data	X	Y	X	Y	X	Y
AVG	4.842	0.431	6.591	−0.601	3.975	1.965
Std. Deviation	0.008	0.010	0.009	0.007	0.008	0.014
Max	4.860	0.460	6.620	−0.590	3.990	1.990
Min	4.820	0.410	6.570	−0.610	3.960	1.930
Diff.	0.04	0.05	0.05	0.02	0.03	0.06
Vertex ID	33		18		19	
Data	X	Y	X	Y	X	Y
AVG	12.696	−0.295	9.486	1.001	7.161	2.003
Std. Deviation	0.011	0.013	0.012	0.013	0.011	0.054
Max	12.720	−0.250	9.510	1.020	7.180	2.120
Min	12.660	−0.320	9.460	0.970	7.150	1.950
Diff.	0.06	0.07	0.05	0.05	0.03	0.17
Vertex ID	14		13		17	
Data	X	Y	X	Y	X	Y
AVG	5.344	2.682	3.693	4.382	9.054	−0.480
Std. Deviation	0.006	0.030	0.013	0.047	0.010	0.014
Max	5.350	2.730	3.720	4.450	9.070	−0.460
Min	5.330	2.620	3.670	4.300	9.030	−0.520
Diff.	0.02	0.11	0.05	0.15	0.04	0.06
Vertex ID	8		4		3	
Data	X	Y	X	Y	X	Y
AVG	7.957	0.766	15.037	−0.423	14.773	−1.055
Std. Deviation	0.012	0.019	0.092	0.039	0.033	0.055
Max	7.990	0.840	15.070	−0.210	14.840	−0.890
Min	7.940	0.740	14.500	−0.470	14.730	−1.210
Diff.	0.05	0.1	0.57	0.26	0.11	0.32
Vertex ID	2		1		31	
Data	X	Y	X	Y	X	Y
AVG	14.165	−2.815	12.957	−5.610	13.078	−2.688
Std. Deviation	0.005	0.009	0.025	0.013	0.035	0.041
Max	14.170	−2.800	13.000	−5.590	13.150	−2.610
Min	14.160	−2.830	12.910	−5.640	13.010	−2.770
Diff.	0.01	0.03	0.09	0.05	0.14	0.16
Vertex ID	7					
Data	X	Y				
AVG	9.863	−0.221				
Std. Deviation	0.013	0.011				
Max	9.890	−0.190				
Min	9.820	−0.240				
Diff.	0.07	0.05				

**Table 8:** Indoor localization system—Qorvo Data.

Vertex ID	5		6		10	
Data	X	Y	X	Y	X	Y
AVG	10.269	−2.818	11.070	−1.371	0.567	−0.165
Std. Deviation	0.235	0.117	0.153	0.124	0.113	0.144
Max	10.897	−2.383	11.741	−1.492	0.742	0.326
Min	10.025	−3.253	10.934	−1.254	0.341	−0.474
Diff.	0.872	0.87	0.807	0.238	0.401	0.8
Vertex ID	12		11		15	
Data	X	Y	X	Y	X	Y
AVG	2.824	4.662	2.214	2.924	3.285	1.605
Std. Deviation	0.036	0.093	0.014	0.025	0.092	0.050
Max	2.978	5.232	2.252	2.992	3.503	1.705
Min	2.753	4.764	2.179	2.857	3.140	1.284
Diff.	0.225	0.468	0.073	0.135	0.363	0.421
Vertex ID	26		16		9	
Data	X	Y	X	Y	X	Y
AVG	5.453	0.881	7.195	−0.020	4.676	2.359
Std. Deviation	0.016	0.034	0.018	0.032	0.081	0.048
Max	5.519	1.083	7.306	0.182	4.782	2.42
Min	5.385	0.787	7.148	−0.266	4.553	2.224
Diff.	0.134	0.296	0.158	0.448	0.229	0.196
Vertex ID	33		18		19	
Data	X	Y	X	Y	X	Y
AVG	12.197	−0.002	9.607	1.294	6.762	2.596
Std. Deviation	0.121	0.148	0.091	0.059	0.052	0.088
Max	12.395	0.285	9.852	1.385	6.854	2.734
Min	11.941	−0.196	9.341	1.213	6.551	2.346
Diff.	0.454	0.481	0.511	0.172	0.303	0.388
Vertex ID	14		13		17	
Data	X	Y	X	Y	X	Y
AVG	5.544	3.475	4.194	4.275	8.555	−0.187
Std. Deviation	0.103	0.095	0.096	0.082	0.045	0.023
Max	5.648	3.546	4.341	4.451	8.594	−0.146
Min	5.384	3.354	4.023	4.123	8.503	−0.321
Diff.	0.264	0.192	0.318	0.328	0.091	0.175
Vertex ID	8		4		3	
Data	X	Y	X	Y	X	Y
AVG	7.858	1.060	14.537	−0.129	13.874	−1.662
Std. Deviation	0.012	0.019	0.042	0.078	0.025	0.031
Max	7.921	1.086	14.795	0.235	14.234	−1.587
Min	7.536	1.042	14.203	−0.421	13.678	−1.753
Diff.	0.385	0.044	0.592	0.656	0.556	0.166
Vertex ID	2		1		31	
Data	X	Y	X	Y	X	Y
AVG	12.966	−3.821	12.457	−4.917	12.579	−2.395
Std. Deviation	0.034	0.017	0.045	0.061	0.036	0.054
Max	13.029	−3.754	12.789	−4.863	12.754	−2.152
Min	12.753	−4.24	12.124	−5.512	12.452	−2.421
Diff.	0.276	0.486	0.665	0.649	0.302	0.269
Vertex ID	7					
Data	X	Y				
AVG	10.164	0.122				
Std. Deviation	0.028	0.036				
Max	10.251	0.156				
Min	10.031	0.063				
Diff.	0.22	0.093				

**Table 9:** Indoor localization system—Marvelmind new points.

Vertex ID	5		6		10	
Data	X	Y	X	Y	X	Y
New Point	1.573	6.317	−0.352	6.386	1.115	−3.945
Vertex ID	12		11		15	
Data	X	Y	X	Y	X	Y
New Point	−3.999	−3.744	−1.720	−3.815	−0.723	−2.485
Vertex ID	26		16		9	
Data	X	Y	X	Y	X	Y
New Point	−0.631	−0.318	−0.556	1.961	−1.818	−1.383
Vertex ID	33		18		19	
Data	X	Y	X	Y	X	Y
New Point	−2.375	7.140	−2.416	3.862	−2.563	1.443
Vertex ID	14		13		17	
Data	X	Y	X	Y	X	Y
New Point	−2.909	−0.475	−3.570	−2.573	−0.673	3.774
Vertex ID	8		4		3	
Data	X	Y	X	Y	X	Y
New Point	−1.742	2.582	−2.922	9.057	−1.808	9.074
Vertex ID	2		1		31	
Data	X	Y	X	Y	X	Y
New Point	0.323	9.021	2.878	8.910	0.156	8.057
Vertex ID	7					
Data	X	Y				
New Point	−1.807	4.660				

**Table 10:** Marvelmind localization system—errors.

Vertex ID	5		6		10	
Data	X	Y	X	Y	X	Y
Diff.	−0.189	−0.005	−0.118	0.027	0.114	0.219
Vertex ID	12		11		15	
Data	X	Y	X	Y	X	Y
Diff.	−0.259	0.296	−0.159	0.276	0.036	0.011
Vertex ID	26		16		9	
Data	X	Y	X	Y	X	Y
Diff.	0.073	−0.150	−0.148	0.102	0.077	−0.015
Vertex ID	33		18		19	
Data	X	Y	X	Y	X	Y
Diff.	0.104	−0.218	0.168	−0.127	0.135	−0.074
Vertex ID	14		13		17	
Data	X	Y	X	Y	X	Y
Diff.	0.055	−0.033	0.063	0.005	−0.163	0.052
Vertex ID	8		4		3	
Data	X	Y	X	Y	X	Y
Diff.	0.154	−0.097	0.423	−0.030	−0.069	−0.006
Vertex ID	2		1		31	
Data	X	Y	X	Y	X	Y
Diff.	−0.124	−0.017	−0.163	−0.021	−0.067	−0.017
Vertex ID	7					
Data	X	Y				
Diff.	0.054	−0.179				

**Table 11:** Indoor localization system—Eliko Kio new points.

Vertex ID	5		6		10	
Data	X	Y	X	Y	X	Y
New Point	1.455	6.283	−0.591	6.493	1.379	−4.180
Vertex ID	12		11		15	
Data	X	Y	X	Y	X	Y
New Point	−4.589	−3.941	−1.867	−4.047	−0.597	−2.975
Vertex ID	26		16		9	
Data	X	Y	X	Y	X	Y
New Point	−0.527	−0.553	−0.080	1.428	−1.722	−1.847
Vertex ID	33		18		19	
Data	X	Y	X	Y	X	Y
New Point	−2.239	7.147	−2.491	3.694	−2.733	1.174
Vertex ID	14		13		17	
Data	X	Y	X	Y	X	Y
New Point	−2.823	−0.764	−3.937	−2.856	−0.949	3.736
Vertex ID	8		4		3	
Data	X	Y	X	Y	X	Y
New Point	−1.799	2.311	−2.834	9.415	−2.151	9.357
Vertex ID	2		1		31	
Data	X	Y	X	Y	X	Y
New Point	−0.290	9.317	2.741	9.022	−0.078	8.243
Vertex ID	7					
Data	X	Y				
New Point	−1.443	4.427				

**Table 12:** Eliko Kio localization system—errors.

Vertex ID	5		6		10	
Data	X	Y	X	Y	X	Y
Diff.	−0.068	−0.036	−0.117	0.132	0.138	−0.010
Vertex ID	12		11		15	
Data	X	Y	X	Y	X	Y
Diff.	−0.609	0.095	−0.066	0.039	0.157	−0.239
Vertex ID	26		16		9	
Data	X	Y	X	Y	X	Y
Diff.	0.176	−0.145	0.322	−0.191	0.165	−0.239
Vertex ID	33		18		19	
Data	X	Y	X	Y	X	Y
Diff.	0.237	0.029	0.092	−0.055	−0.040	−0.103
Vertex ID	14		13		17	
Data	X	Y	X	Y	X	Y
Diff.	0.129	−0.082	−0.306	−0.038	−0.199	0.012
Vertex ID	8		4		3	
Data	X	Y	X	Y	X	Y
Diff.	0.093	−0.128	0.271	0.328	−0.172	0.272
Vertex ID	2		1		31	
Data	X	Y	X	Y	X	Y
Diff.	−0.497	0.274	−0.060	0.086	−0.061	0.171
Vertex ID	7					
Data	X	Y				
Diff.	0.412	−0.171				

**Table 13:** Indoor localization system—Qorvo new points.

Vertex ID	5		6		10	
Data	X	Y	X	Y	X	Y
New Point	1.099	5.979	−0.531	6.259	1.793	−4.055
Vertex ID	12		11		15	
Data	X	Y	X	Y	X	Y
New Point	−3.508	−3.516	−1.666	−3.518	−0.774	−2.072
Vertex ID	26		16		9	
Data	X	Y	X	Y	X	Y
New Point	−0.805	0.213	−0.529	2.155	−1.945	−1.008
Vertex ID	33		18		19	
Data	X	Y	X	Y	X	Y
New Point	−2.195	6.871	−2.565	3.999	−2.856	0.884
Vertex ID	14		13		17	
Data	X	Y	X	Y	X	Y
New Point	−3.284	−0.556	−3.595	−2.095	−0.820	3.494
Vertex ID	8		4		3	
Data	X	Y	X	Y	X	Y
New Point	−1.767	2.425	−2.847	9.122	−1.181	9.002
Vertex ID	2		1		31	
Data	X	Y	X	Y	X	Y
New Point	1.157	8.856	2.359	8.737	−0.062	8.021
Vertex ID	7					
Data	X	Y				
New Point	−1.642	4.911				

**Table 14:** Qorvo localization system—errors.

Vertex ID	5		6		10	
Data	X	Y	X	Y	X	Y
Diff.	−0.718	−0.343	−0.352	−0.100	0.847	0.108
Vertex ID	12		11		15	
Data	X	Y	X	Y	X	Y
Diff.	0.177	0.525	−0.160	0.574	−0.014	0.369
Vertex ID	26		16		9	
Data	X	Y	X	Y	X	Y
Diff.	−0.100	0.326	−0.120	0.241	−0.048	0.305
Vertex ID	33		18		19	
Data	X	Y	X	Y	X	Y
Diff.	0.285	−0.542	0.020	−0.045	−0.157	−0.689
Vertex ID	14		13		17	
Data	X	Y	X	Y	X	Y
Diff.	−0.317	−0.169	0.040	0.429	−0.365	−0.228
Vertex ID	8		4		3	
Data	X	Y	X	Y	X	Y
Diff.	0.130	−0.308	0.553	0.035	0.503	−0.077
Vertex ID	2		1		31	
Data	X	Y	X	Y	X	Y
Diff.	0.655	−0.181	−0.737	−0.193	−0.340	−0.054
Vertex ID	7					
Data	X	Y				
Diff.	0.220	0.018				

**Table 15:** Indoor localization systems—error points comparison.

Vertex ID	5		6		10	
Localization Systems	X	Y	X	Y	X	Y
Marvelmind	−0.189	−0.005	−0.118	0.027	0.114	0.219
Qorvo	−0.718	−0.343	−0.352	−0.100	0.847	0.108
Eliko Kio	−0.068	−0.036	−0.117	0.132	0.138	−0.010
Vertex ID	12		11		15	
Localization Systems	X	Y	X	Y	X	Y
Marvelmind	−0.259	0.296	−0.159	0.276	0.036	0.011
Qorvo	0.177	0.525	−0.160	0.574	−0.014	0.369
Eliko Kio	−0.609	0.095	−0.066	0.039	0.157	−0.239
Vertex ID	26		16		9	
Localization Systems	X	Y	X	Y	X	Y
Marvelmind	0.073	−0.150	−0.148	0.102	0.077	−0.015
Qorvo	−0.100	0.326	−0.120	0.241	−0.048	0.305
Eliko Kio	0.176	−0.145	0.322	−0.191	0.165	−0.239
Vertex ID	33		18		19	
Localization Systems	X	Y	X	Y	X	Y
Marvelmind	0.104	−0.218	0.168	−0.127	0.135	−0.074
Qorvo	0.285	−0.542	0.020	−0.045	−0.157	−0.689
Eliko Kio	0.237	0.029	0.092	−0.055	−0.040	−0.103
Vertex ID	14		13		17	
Localization Systems	X	Y	X	Y	X	Y
Marvelmind	0.055	−0.033	0.063	0.005	−0.163	0.052
Qorvo	−0.317	−0.169	0.040	0.429	−0.365	−0.228
Eliko Kio	0.129	−0.082	−0.306	−0.038	−0.199	0.012
Vertex ID	8		4		3	
Localization Systems	X	Y	X	Y	X	Y
Marvelmind	0.154	−0.097	0.423	−0.030	−0.069	−0.006
Qorvo	0.130	−0.308	0.553	0.035	0.503	−0.077
Eliko Kio	0.093	−0.128	0.271	0.328	−0.172	0.272
Vertex ID	2		1		31	
Localization Systems	X	Y	X	Y	X	Y
Marvelmind	−0.124	−0.017	−0.163	−0.021	−0.067	−0.017
Qorvo	0.655	−0.181	−0.737	−0.193	−0.340	−0.054
Eliko Kio	−0.497	0.274	−0.060	0.086	−0.061	0.171
Vertex ID	7					
Localization Systems	X	Y				
Marvelmind	0.054	−0.179				
Qorvo	0.220	0.018				
Eliko Kio	0.412	−0.171				

**Table 16:** Euclidean distances to ground truth System.

Vertex ID	5		6		10	
**Localization** **Systems**	X	Y	X	Y	X	Y
Marvelmind	0.189		**0.121**		0.247	
Qorvo	0.796		0.366		0.854	
Eliko Kio	**0.077**		0.176		**0.138**	
Vertex ID	12		11		15	
**Localization** **Systems**	X	Y	X	Y	X	Y
Marvelmind	**0.393**		0.318		**0.038**	
Qorvo	0.554		0.596		0.369	
Eliko Kio	0.616		**0.077**		0.286	
Vertex ID	26		16		9	
**Localization** **Systems**	X	Y	X	Y	X	Y
Marvelmind	**0.167**		**0.180**		**0.078**	
Qorvo	0.341		0.269		0.309	
Eliko Kio	0.228		0.374		0.290	
Vertex ID	33		18		19	
**Localization** **Systems**	X	Y	X	Y	X	Y
Marvelmind	0.242		0.211		0.154	
Qorvo	0.612		**0.049**		0.707	
Eliko Kio	**0.239**		0.107		**0.110**	
Vertex ID	14		13		17	
**Localization** **Systems**	X	Y	X	Y	X	Y
Marvelmind	**0.064**		**0.063**		**0.171**	
Qorvo	0.359		0.431		0.430	
Eliko Kio	0.153		0.308		0.199	
Vertex ID	8		4		3	
**Localization** **Systems**	X	Y	X	Y	X	Y
Marvelmind	0.182		**0.424**		**0.069**	
Qorvo	0.334		0.554		0.509	
Eliko Kio	**0.158**		0.425		0.322	
Vertex ID	2		1		31	
**Localization** **Systems**	X	Y	X	Y	X	Y
Marvelmind	**0.125**		0.164		**0.069**	
Qorvo	0.679		0.762		0.344	
Eliko Kio	0.567		**0.105**		0.182	
Vertex ID	7					
**Localization** **Systems**	X	Y				
Marvelmind	**0.187**					
Qorvo	0.221					
Eliko Kio	0.446					

## Data Availability

This study did not report any new data.

## Abbreviations

The following abbreviations are used in this manuscript:

AGV	Autonomous Guided Vehicle
AMR	Autonomous Mobile Robots
AoA	Angle of Arrival
API	Application Programming Interface
BLE	Bluetooth Low Energy
EKF	Extended Kalman Filter
EU	European Union
GPS	Global Positioning System
ID	Identification Number
IMUs	Inertial Measurement Units
IoT	Internet of Things
IR	Infrared
LS	Least Squares
MDPI	Multidisciplinary Digital Publishing Institute
ML	Machine Learning
MP	Mobile Platform
RF	Radio Frequency
RFID	Radio Frequency IDentification
RSS	Received Signal Strength
RSSI	Received Signal Strength Indicator
RVIZ	Robot Operating System Visualization
TDoA	Time Difference of Arrival
TEA*	Time Enhanced A*
3D	Three-Dimensional
ToA	Time of Arrival
ToF	Time of Flight
2D	Two-Dimensional
UHF	Ultra High Frequency
US	Ultrasound
UWB	Ultra-Wideband
WLAN	Wireless Local Area Network

## References

[1] Moura P., Costa P., Lima J., Costa P. (2019). A temporal optimization applied to time enhanced A. AIP Conference Proceedings.

[2] Santos J., Costa P., Rocha L.F., Moreira A.P., Veiga G. Time enhanced A*: Towards the development of a new approach for Multi-Robot Coordination. Proceedings of the 2015 IEEE International Conference on Industrial Technology (ICIT).

[3] Cardarelli E., Digani V., Sabattini L., Secchi C., Fantuzzi C. (2017). Cooperative cloud robotics architecture for the coordination of multi-AGV systems in industrial warehouses. Mechatronics.

[4] Butdee S., Suebsomran A. Localization based on matching location of AGV. Proceedings of the 24th International Manufacturing Conference, IMC24. Waterford Institute of Technology.

[5] Roy P., Chowdhury C. (2021). A survey of machine learning techniques for indoor localization and navigation systems. J. Intell. Robot. Syst..

[6] Zafari F., Gkelias A., Leung K.K. (2019). A survey of indoor localization systems and technologies. IEEE Commun. Surv. Tutor..

[7] Bradley C., El-Tawab S., Heydari M.H. Security analysis of an IoT system used for indoor localization in healthcare facilities. Proceedings of the 2018 Systems and Information Engineering Design Symposium (SIEDS).

[8] Shit R.C., Sharma S., Yelamarthi K., Puthal D. (2021). AI-enabled fingerprinting and crowdsource-based vehicle localization for resilient and safe transportation systems. IEEE Trans. Intell. Transp. Syst..

[9] Obeidat H., Shuaieb W., Obeidat O., Abd-Alhameed R. (2021). A review of indoor localization techniques and wireless technologies. Wirel. Pers. Commun..

[10] Pilati F., Sbaragli A., Nardello M., Santoro L., Fontanelli D., Brunelli D. (2022). Indoor positioning systems to prevent the COVID19 transmission in manufacturing environments. Procedia Cirp.

[11] Xiong R., van Waasen S., Rheinlnder C., Wehn N. (2017). Development of a Novel Indoor Positioning System With mm-Range Precision Based on RF Sensors Network. IEEE Sens. Lett..

[12] Li N., Becerik-Gerber B. An infrastructure-free indoor localization framework to support building emergency response operations. Proceedings of the 19th EG-ICE International Workshop on Intelligent Computing in Engineering.

[13] Wang S., Zhao L. Optimization of Goods Location Numbering and Storage and Retrieval Sequence in Automated Warehouse. Proceedings of the 2009 International Joint Conference on Computational Sciences and Optimization.

[14] Lipka M., Sippel E., Hehn M., Adametz J., Vossiek M., Dobrev Y., Gulden P. Wireless 3D Localization Concept for Industrial Automation Based on a Bearings Only Extended Kalman Filter. Proceedings of the 2018 Asia-Pacific Microwave Conference (APMC).

[15] Hesslein N., Wesselhöft M., Hinckeldeyn J., Kreutzfeldt J. (2021). Industrial indoor localization: Improvement of logistics processes using location based services. Advances in Automotive Production Technology–Theory and Application: Stuttgart Conference on Automotive Production (SCAP2020).

[16] Xu L., Shen X., Han T.X., Du R., Shen Y. An Efficient Relative Localization Method via Geometry-based Coordinate System Selection. Proceedings of the ICC 2022-IEEE International Conference on Communications.

[17] Luo Q., Yang K., Yan X., Li J., Wang C., Zhou Z. (2022). An Improved Trilateration Positioning Algorithm with Anchor Node Combination and K-Means Clustering. Sensors.

[18] Thrun S., Burgard W., Fox D. (2005). Probabilistic Robotics (Intelligent Robotics and Autonomous Agents).

[19] Kim S.H., Roh C.W., Kang S.C., Park M.Y. Outdoor navigation of a mobile robot using differential GPS and curb detection. Proceedings of the 2007 IEEE International Conference on Robotics and Automation.

[20] Gonzalez J., Blanco J., Galindo C., Ortiz-de Galisteo A., Fernández-Madrigal J., Moreno F., Martinez J. Combination of UWB and GPS for indoor-outdoor vehicle localization. Proceedings of the 2007 IEEE International Symposium on Intelligent Signal Processing.

[21] Hahnel D., Burgard W., Fox D., Fishkin K., Philipose M. Mapping and localization with RFID technology. Proceedings of the IEEE International Conference on Robotics and Automation, 2004. Proceedings. ICRA’04.

[22] Choi B.S., Lee J.W., Lee J.J., Park K.T. (2011). A hierarchical algorithm for indoor mobile robot localization using RFID sensor fusion. IEEE Trans. Ind. Electron..

[23] Huh J., Chung W.S., Nam S.Y., Chung W.K. (2007). Mobile robot exploration in indoor environment using topological structure with invisible barcodes. ETRI J..

[24] Lin G., Chen X. (2011). A Robot Indoor Position and Orientation Method based on 2D Barcode Landmark. J. Comput..

[25] Kobayashi H. A new proposal for self-localization of mobile robot by self-contained 2d barcode landmark. Proceedings of the 2012 Proceedings of SICE annual conference (SICE).

[26] Atanasyan A., Roßmann J. (2019). Improving Self-Localization Using CNN-based Monocular Landmark Detection and Distance Estimation in Virtual Testbeds. Tagungsband des 4. Kongresses Montage Handhabung Industrieroboter.

[27] Kendall A., Grimes M., Cipolla R. Posenet: A convolutional network for real-time 6-dof camera relocalization. Proceedings of the IEEE International Conference on Computer Vision.

[28] Sadeghi Esfahlani S., Sanaei A., Ghorabian M., Shirvani H. (2022). The Deep Convolutional Neural Network Role in the Autonomous Navigation of Mobile Robots (SROBO). Remote Sens..

[29] Szegedy C., Liu W., Jia Y., Sermanet P., Reed S., Anguelov D., Erhan D., Vanhoucke V., Rabinovich A. Going deeper with convolutions. Proceedings of the 2015 IEEE Conference on Computer Vision and Pattern Recognition (CVPR).

[30] Lima J., Rocha C., Rocha L., Costa P. (2022). Data Matrix Based Low Cost Autonomous Detection of Medicine Packages. Appl. Sci..

[31] Sharma P., Saucan A.A., Bucci D.J., Varshney P.K. On Self-Localization and Tracking with an Unknown Number of Targets. Proceedings of the 2018 52nd Asilomar Conference on Signals, Systems, and Computers.

[32] Ahmad U., Poon K., Altayyari A.M., Almazrouei M.R. A Low-cost Localization System for Warehouse Inventory Management. Proceedings of the 2019 International Conference on Electrical and Computing Technologies and Applications (ICECTA).

[33] Halawa F., Dauod H., Lee I.G., Li Y., Yoon S.W., Chung S. (2020). Introduction of a real time location system to enhance the warehouse safety and operational efficiency. Int. J. Prod. Econ..

[34] Coronado E., Kiyokawa T., Ricardez G.A.G., Ramirez-Alpizar I.G., Venture G., Yamanobe N. (2022). Evaluating quality in human-robot interaction: A systematic search and classification of performance and human-centered factors, measures and metrics towards an industry 5.0. J. Manuf. Syst..

[35] Martinho R., Lopes J., Jorge D., de Oliveira L.C., Henriques C., Peças P. (2022). IoT Based Automatic Diagnosis for Continuous Improvement. Sustainability.

[36] Le D.V., Havinga P.J. SoLoc: Self-organizing indoor localization for unstructured and dynamic environments. Proceedings of the 2017 International Conference on Indoor Positioning and Indoor Navigation (IPIN).

[37] Flögel D., Bhatt N.P., Hashemi E. (2022). Infrastructure-Aided Localization and State Estimation for Autonomous Mobile Robots. Robotics.

[38] Alkendi Y., Seneviratne L., Zweiri Y. (2021). State of the Art in Vision-Based Localization Techniques for Autonomous Navigation Systems. IEEE Access.

[39] Dias F., Schafer H., Natal L., Cardeira C. Mobile Robot Localisation for Indoor Environments Based on Ceiling Pattern Recognition. Proceedings of the 2015 IEEE International Conference on Autonomous Robot Systems and Competitions.

[40] Sudin M., Abdullah S., Nasudin M. (2019). Humanoid Localization on Robocup Field using Corner Intersection and Geometric Distance Estimation. IJIMAI.

[41] Kalaitzakis M., Cain B., Carroll S., Ambrosi A., Whitehead C., Vitzilaios N. (2021). Fiducial markers for pose estimation. J. Intell. Robot. Syst..

[42] Grilo A., Costa R., Figueiras P., Gonçalves R.J. Analysis of AGV indoor tracking supported by IMU sensors in intra-logistics process in automotive industry. Proceedings of the 2021 IEEE International Conference on Engineering, Technology and Innovation (ICE/ITMC).

[43] Malyavej V., Kumkeaw W., Aorpimai M. Indoor robot localization by RSSI/IMU sensor fusion. Proceedings of the 2013 10th International Conference on Electrical Engineering/Electronics, Computer, Telecommunications and Information Technology.

[44] Xia Z., Chen C. A Localization Scheme with Mobile Beacon for Wireless Sensor Networks. Proceedings of the 2006 6th International Conference on ITS Telecommunications.

[45] Zhao C., Wang B. A UWB/Bluetooth Fusion Algorithm for Indoor Localization. Proceedings of the 2019 Chinese Control Conference (CCC).

[46] Álvarez Merino C.S., Luo-Chen H.Q., Khatib E.J., Barco R. (2021). WiFi FTM, UWB and Cellular-Based Radio Fusion for Indoor Positioning. Sensors.

[47] Zhang L., Wu X., Gao R., Pan L., Zhang Q. (2023). A multi-sensor fusion positioning approach for indoor mobile robot using factor graph. Measurement.

[48] Dargie W., Poellabauer C. (2010). Fundamentals of Wireless Sensor Networks: Theory and Practice.

[49] Xiong J., Jamieson K. ArrayTrack: A Fine-Grained indoor location system. Proceedings of the 10th USENIX Symposium on Networked Systems Design and Implementation (NSDI 13).

[50] Liu H., Darabi H., Banerjee P., Liu J. (2007). Survey of wireless indoor positioning techniques and systems. IEEE Trans. Syst. Man Cybern. Part C.

[51] Oppermann I., Hämäläinen M., Iinatti J. (2004). UWB: Theory and Applications.

[52] Ijaz F., Yang H.K., Ahmad A.W., Lee C. Indoor positioning: A review of indoor ultrasonic positioning systems. Proceedings of the 2013 15th International Conference on Advanced Communications Technology (ICACT).

[53] Qorvo (2024). Qorvo All Around You. https://www.qorvo.com/.

[54] Pozyx (2024). Pozyx. https://www.pozyx.io/.

[55] Eliko (2024). Next-Generation Location Tracking. https://eliko.tech/.

[56] Marvelmind (2023). Marvelmind Robotics. https://marvelmind.com/.

